# Epidermal radionuclide therapy with rhenium-188 in non-melanoma skin cancer: a short narrative review

**DOI:** 10.3389/fmed.2026.1850326

**Published:** 2026-06-03

**Authors:** Paolo Castellucci, Luigia Vetrone, Carlotta Baraldi, Emi Dika, Federico Zagni, Lidia Strigari, Elisa Martínez Albero, Diana Vega Pérez, Siroos Mirzaei, Stefano Fanti

**Affiliations:** 1 Nuclear Medicine IRCCS Azienda Ospedaliero-Universitaria S.Orsola, Bologna, Italy; 2 Oncologic Dermatology Unit, IRCCS Azienda Ospedaliero-Universitaria S.Orsola, Bologna, Italy; 3 Department of Medical Physics, IRCCS Azienda Ospedaliero-Universitaria di Bologna, Bologna, Italy; 4 Medicina Nuclear Hospital Universitario 12 de Octubre, Madrid, Spain; 5 Department of Nuclear Medicine with PET-Centre, Clinic Ottakring, Vienna, Austria

**Keywords:** brachytherapy, epidermal radionuclide therapy, non-melanoma skin cancer, radionuclide treatment, rhenium-188

## Abstract

**Background:**

Non-melanoma skin cancer (NMSC), represents the most common malignancy in fair-skinned populations and constitutes a growing public health burden. Although surgery remains the standard of care, a significant proportion of patients are poor surgical candidates due to age, comorbidities, tumor location or personal preference, highlighting the need for effective non-surgical alternatives.

**Objective:**

To review current therapeutic strategies and the clinical, physical and radiobiological basis, indications, efficacy and safety of rhenium-188 Epidermal Radionuclide Therapy (ERT) as an emerging treatment option in NMSC.

**Results:**

Re-188 ERT is a form of high-dose superficial brachytherapy using an unsealed beta-emitting radioisotope embedded in a synthetic resin applied directly to the skin surface. Owing to the limited penetration depth of beta radiation, high doses can be delivered selectively to superficial tumors while sparing deeper tissues. Clinical series report complete response rates ranging from 90 to 100%, with very low recurrence rates and predominantly mild, transient toxicity. Cosmetic outcomes are generally good to excellent. Emerging evidence also supports its potential role in extramammary Paget disease and refractory keloids.

**Conclusion:**

Re-188 ERT is a safe, effective and minimally invasive alternative for selected patients with NMSC, particularly those unsuitable for surgery or with tumours in anatomically complex areas. While it does not replace surgery as first-line treatment, its high efficacy, favourable cosmetic outcomes and single-session outpatient delivery makes it a valuable addition to the therapeutic armamentarium. Further prospective studies are warranted to optimize dosimetry, reduce toxicity and expand evidence for additional indications.

## Introduction

Non-melanoma skin cancer (NMSC), is the most prevalent malignancy in fair-skinned populations worldwide (1, 2). Across Europe, approximately 27.1 million NMSC cases were registered between 1992 and 2021, with Basal Cell Carcinoma (BCC) accounting for 86% and cutaneous Squamous Cell Carcinoma (cSCC) for 14% (1, 2). NMSC is associated with relatively low mortality but represents a significant healthcare burden due to its high incidence, potential for local tissue destruction and, in the case of cSCC, risk of metastasis and disease-related mortality, especially in elderly and immunosuppressed patients (1, 2).

Management of NMSC is highly individualized and depends on tumour type, histological subtype, depth of invasion, anatomical location and patient-related factors, Surgical excision, however, is the first-line treatment for most NMSC cases, achieving cure rates exceeding 90% in low-risk BCC (3). Mohs micrographic surgery provides the highest cure rates (>98%) for high-risk or recurrent tumors (4). However, a substantial subgroup of patients are not ideal surgical candidates due to age, comorbidities, anatomical constraints or personal preference (3). This has driven interest in alternative, minimally invasive therapeutic approaches: cryotherapy, curettage with electrodessication and laser therapy, are suitable for selected low-risk lesions but lack histological margin control and are operator dependent; Laser-based approaches, such as Nd: YAG laser therapy, have shown low recurrence rates in selected cohorts (5); imiquimod 5% and 5-fluorouracil cream, are used in superficial BCC and Bowen’s disease, with long-term control rates around 80% in well-selected cases, though with lower efficacy than surgery and frequent local inflammatory reactions (6); Photodynamic therapy (PDT) offers excellent cosmetic outcomes and good short-term response rates but is associated with higher long-term recurrence compared with surgery or topical immunotherapy in thicker or extensive lesions (6). Radiotherapy (RT), including external beam RT, brachytherapy and image-guided superficial RT, plays a key role in elderly or frail patients, in inoperable tumors and as adjuvant therapy for high-risk cSCC. RT provides durable local control but requires multiple fractions and may lead to chronic skin toxicity (7, 8).

The use of radioisotopes in dermatologic oncology dates back to the 1960s with encapsulated sources such as iridium-192 (7, 8). Although conventional radiotherapy achieves cure rates close to 90%, it often requires complex planning and multiple treatment sessions, which may be impractical for some patients. Early experimental and clinical studies with superficial brachytherapy using unsealed beta-emitting radioisotopes, Epidermal Radionuclide Therapy (ERT) particularly rhenium-188 (Re-188) resin, has emerged as a promising option (9–11). Building on this concept, Re-188 ERT was developed as a high-dose superficial radiotherapy technique allowing homogeneous dose deposition limited to the tumour depth. Re-188 ERT consists of a synthetic, inert resin containing unsealed Re-188 applied directly to the lesion surface. The technique enables precise dose delivery while sparing deeper tissues and surrounding healthy skin. This review summarizes the clinical, physical and radiobiological foundations, and the efficacy and safety of Re-188 ERT according to the data published so far. Given the small number of publications, the application of a formal systematic review framework, would not have provided additional value or meaningful differentiation among studies. Instead, we focused on a comprehensive and critical appraisal of the existing evidence.

### Physical and radiobiological principles

Re-188 is a *β*−/*γ*-emitting radioisotope with a physical half-life of 17 h, decaying to stable osmium-188. Because of the limited penetration of its beta emissions in tissue, ^188^Re is particularly suitable for the treatment of superficial skin tumours. Most of the absorbed dose is delivered within the first few millimetres from the application surface, with a steep dose fall-off with depth, allowing high local dose deposition in the tumour while relatively sparing deeper healthy tissues. Besides, the gamma emission has little relevance for the local therapeutic effect, which is mainly driven by beta particle. The antitumor effect is mediated by both direct and indirect mechanisms. Direct effects include beta radiation–induced DNA damage leading predominantly to single-strand breaks and apoptotic cell death. Indirect effects arise from the generation of reactive oxygen species, bystander effects on adjacent non-irradiated cells, local immune activation and tumor hypovascularization, contributing to effective tumor control while preserving deeper dermal structures (12, 13). However, the actual dose distribution is strongly influenced by lesion thickness, treated surface area, anatomical irregularities, and the quality and uniformity of resin application, highlighting the importance of dedicated and patient-specific dosimetric approaches.

### Indications

Current indications for Re-188 ERT (14) include BCC and cSCC with an invasion depth ≤3 mm and no evidence of perineural involvement. Patient selection should take into account well-defined, less aggressive lesions. Uncertainty in lesion margins may reduce treatment effectiveness, while infiltrative or high-risk tumors should generally be excluded from treatment with Re-188. This modality is particularly appropriate in the following clinical scenarios:

Elderly patients or individuals with significant comorbidities who are not suitable candidates for surgery; Tumors located in anatomically complex or cosmetically sensitive areas, where surgical intervention may compromise function or aesthetic outcomes; Recurrent or treatment-refractory lesions; Patients with multiple lesions, for whom a surgical approach may be impractical or overly burdensome; there is no strict upper limit to the surface area that can be treated with Re-188, however, increasing treatment extent is associated with a higher incidence of severe adverse events and a greater likelihood of relapse, particularly at the lesion margins.

**Figure 1:**
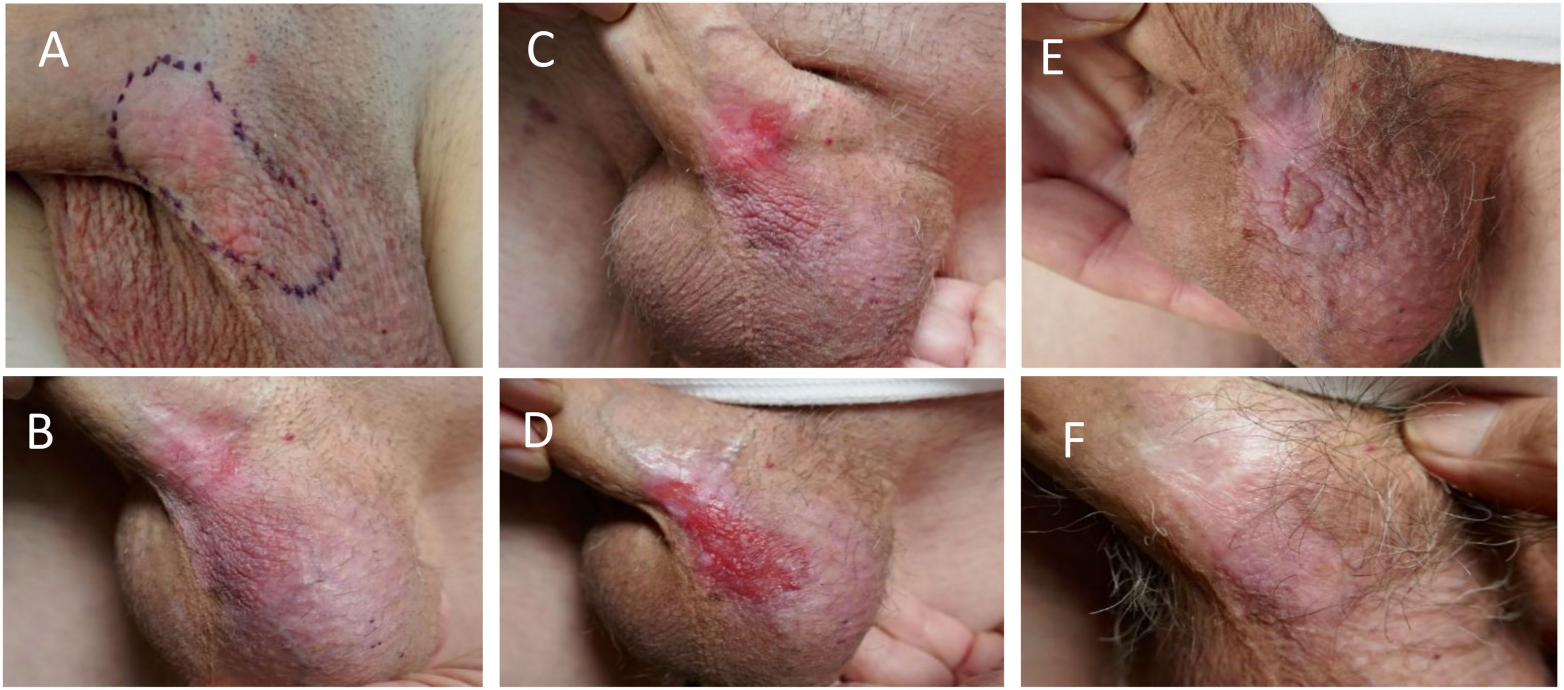
72-year-old patient (M) with extra mammary Paget disease. Surface area 12.5 cm^2^; thickness 1.5 mm **(A)** Before Re-188 ERT; **(B)** after 7 days; **(C)** 20 days; **(D)** 30 days; **(E)** 45 days; **(F)** 90 days showing a complete clinical response and very faint depigmentation.

**Figure 2:**
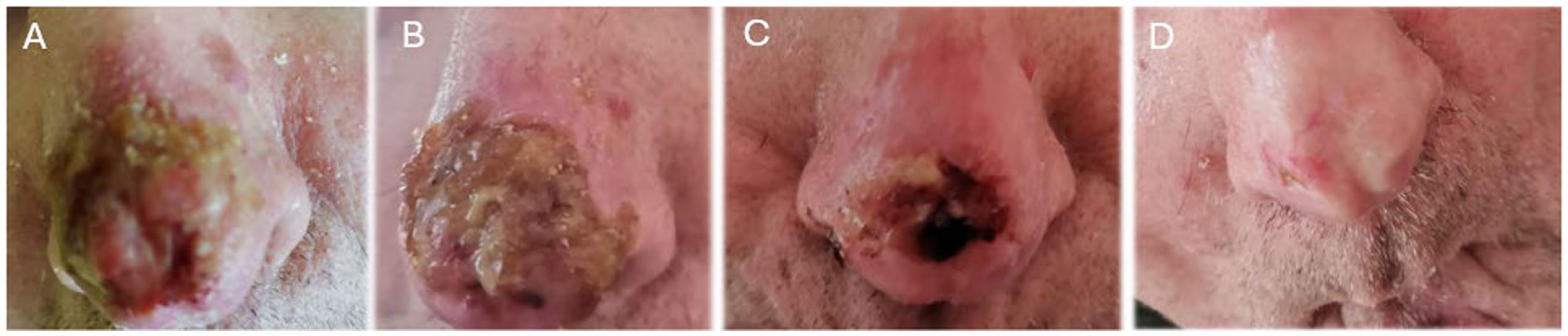
83 year old patient (M) with dementia, paraplegia, and BCC with tumor depth > 3 mm: **(A)** Before Re-188 ERT; **(B)** after 4 weeks; **(C)** 8 weeks; **(D)** 4 months showing reasonable clinical response. The procedure was performed with palliative intent.

Emerging indications include extra mammary Paget disease (as shown in Figure 1), or cases of advanced lesions in whom only palliative treatments are feasible, but where Re-188 ERT can still provide excellent results (as shown in Figure 2), Actinic Keratosis and refractory keloids, where early reports suggest favourable response rates and acceptable toxicity profiles (15–17).

### Efficacy, safety and cosmetic outcomes

Given the limited and heterogeneous nature of the available evidence base, and the lack of randomized controlled trials. The reported efficacy, safety and cosmetic outcomes should be interpreted with caution. Published clinical series report high complete response (CR) rates with Re-188 epidermal radionuclide therapy in NMSC, with very low recurrence rates in most series. Overall, response rates typically range from 80 to 100%, with better outcomes observed in lesions with treated surface areas generally below ~8–10 cm^2^ (18–21). Larger surface areas are more frequently associated with lower remission rates and a higher likelihood of retreatment. A recent single-center, study by Castellucci et al. (22) evaluating Re-188 ERT in 124 patients with 181 NMSC lesions, demonstrated a high relapse-free rate of 91.1% after a median follow up of 36 months, with most relapses occurring in larger lesions and in the lesions’ margins, supporting the observation that smaller lesions (<8 cm^2^) achieve better long-term control (96.5% relapse-free after 12 months follow up). In this series early adverse reactions were predominantly mild (grade 1–2) according to the CTCAE v 5.0 (23) and resolved within approximately one month; severe reactions (grade 3) were uncommon and, however, resolved within 90 days.

The *EPIC-Skin* trial (24), a prospective, international, multicenter, phase IV study, provides some of the largest prospective clinical evidence for Re-188 ERT. In the 6-month interim analysis, 97.2% (103/106) of evaluated tumors achieved complete response using modified RECIST criteria (25), with 2.8% (3/106) partial responses; no pain or discomfort during treatment was reported, and patient-reported quality of life improved significantly (EPIC-Skin). Adverse events at 6 months occurred in 15.9% of patients and were mostly grade 1–2, with a single grade 3 event noted.

In the 12-month interim analysis from the *EPIC-Skin* dataset (26), which assessed 185 treated lesions across 140 patients, 94.1% (174/185) achieved complete response and 3.2% (6/185) partial response at 12 months, with the remainder classified as stable or progressive disease. Quality of life improvements were sustained, and treatment comfort and cosmetic outcomes remained favorable. Radiation dermatitis was common but mostly CTCAE grade 1–2 and resolved rapidly. The most frequent 12-month toxicity was grade 1 hypopigmentation (≈60%), with no grade 3 or 4 toxicities observed at this time point. Patient- and clinician-reported cosmesis scores at 12 months were high on a 10-point scale, indicating broadly favorable aesthetic results.

**Figure 3:**
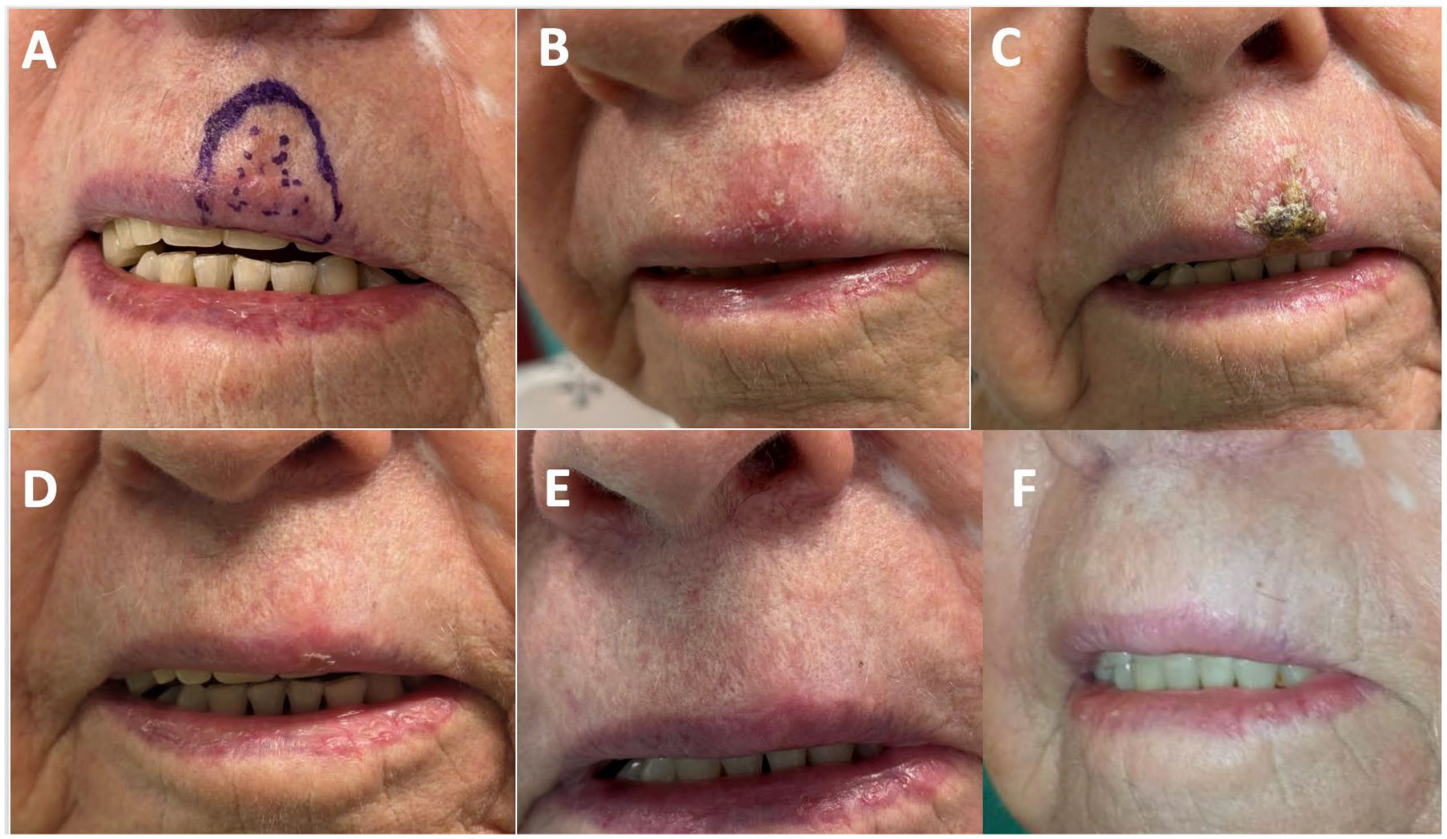
91-year-old female with infiltrative basal cell carcinoma in upper lip; surface area 3.83 cm^2^; thickness 1 mm. Target dose 30 Gy. **(A)** Before Re-188 ERT; **(B)** 15 days after treatment showed mild erythema; **(C)** after 30 days we observe a crust; **(D)** 2 months post-treatment; **(E)** 3 months post-treatment; **(F)** after 4 months showing a complete clinical response and excellent cosmetic result without any changes in pigmentation.

Cosmetic outcomes are predominantly good to excellent, according to the Radiation Therapy Oncology Group (RTOG) and the European Organization for Research and Treatment of Cancer (EORTC) criteria (27), particularly for lesions on the head and face (as shown in Figure 3). Hypopigmentation remains the most frequent late effect, reported in a substantial proportion of treated lesions (≈49–60%), without significant functional impairment (21, 23, 26). Patient satisfaction and quality-of-life measures improve significantly after treatment, with the EPIC-Skin study demonstrating meaningful gains in validated skin-cancer-specific quality-of-life scores.

## Discussion

Re-188 epidermal radionuclide therapy fills a therapeutic gap in the management of non-melanoma skin cancer (NMSC), particularly for patients who are poor surgical candidates due to advanced age, comorbidities, number or location of the lesions, as well as for those who decline surgery because of concerns related to functional impairment, cosmetic outcomes or risk associated with the procedure (28, 29). It’s single-session outpatient administration, minimal invasiveness, and absence of anesthesia requirements, make Re-188 ERT especially attractive in frail or elderly populations. Moreover, the ability to deliver a high, spatially confined radiation dose to superficial tumours while sparing deeper tissues translates into high tumour control rates, low recurrence, and generally favourable cosmetic outcomes, particularly in anatomically sensitive areas such as the face and scalp. Despite these encouraging clinical results, the current body of evidence supporting Re-188 ERT is still largely derived from observational studies, single-center experiences and relatively small patient cohorts. While prospective data such as those from the EPIC-Skin trial (24) and long-term follow-up reported by Castellucci et al. (22) provide robust signals of efficacy and safety, randomized comparative trials against standard treatments (surgery, topical therapies or conventional radiotherapy) remain lacking. As a result, the precise positioning of Re-188 ERT within clinical guidelines has yet to be fully established. Future research should focus on several key areas:

- Dosimetry is typically estimated using simplified models based on lesion thickness and treated area; however, real dose distribution may be influenced by anatomical factors and resin application, and remains insufficiently standardized. The doses delivered differ from study to study. Approximately, they range from 25 to 50 Gy to the deepest point of invasion. However, this can also depend on the size, thickness and location of the lesions, so a “one fits all” standardized dose applicable to all lesions has not yet been established. Dosimetry optimization is needed to better individualize treatment, particularly in relation to lesion depth and treated surface area, which have been consistently associated with both efficacy and toxicity. In this context, established computational approaches for skin dose assessment, such as VARSKIN (30), may offer a useful conceptual framework for superficial beta dosimetry, although dedicated models are required for the specific geometry and source distribution of Re-188 resin applications (31).
- Dose de-escalation strategies deserve investigation in smaller or superficial lesions to minimize acute toxicity and late cosmetic changes without compromising tumour control. Standardized protocols for retreatment in cases of partial response, persistence or relapse (including the timing and number of additional applications), should be further evaluated in future studies as re-treatment has shown excellent efficacy in few published series.
- Finally, longer follow-up and broader inclusion criteria are required to define long-term outcomes, assess durability of response, and explore expanded indications, including Extra mammary Paget disease, other superficial cutaneous malignancies, the use as a palliative therapy and finally the application in refractory keloids. In summary, Re-188 ERT represents a valuable addition to the range of therapies for NMSC, offering a patient-centered, effective and cosmetically favourable alternative when surgery is contraindicated or declined. Continued prospective research will be essential to consolidate its role, refine treatment protocols and support its integration into routine clinical practice.

## Conclusion

Re-188 epidermal radionuclide therapy has emerged as a safe, effective and minimally invasive treatment option for selected patients with non-melanoma skin cancer. Although surgery remains the standard of care, Re-188 ERT provides high rates of durable tumour control, low recurrence, and consistently favourable cosmetic outcomes when applied in appropriately selected superficial lesions, particularly in patients who are unfit for or decline surgical intervention. Its single-session outpatient delivery, favorable tolerability profile and patient-reported quality-of-life benefits underscore its value as a patient-centered therapeutic alternative. Continued accumulation of real-world data and comparative trials will be essential to define the precise positioning of Re-188 ERT within clinical algorithms and to support its broader integration into routine practice. In parallel, expanding clinical experience may establish its role in additional indications, further extending the impact of this radio nuclide therapy in dermatologic oncology.
